# Understanding the Experiences of Adolescents and Young Adults Diagnosed with Cancer During Higher Education—An Exploratory Interview Study

**DOI:** 10.3390/cancers18020325

**Published:** 2026-01-20

**Authors:** Anke W. Boumans, Margo J. van Hartingsveldt, Angela G. E. M. de Boer, Maaike M. Brus, Floor M. Hoddenbagh-Bosdijk, Milou J. P. Reuvers, Jack D. Morgan, Martijn M. Stuiver, Olga Husson

**Affiliations:** 1 Department of Occupational Therapy and Centre of Expertise Urban Vitality, Amsterdam University of Applied Sciences, 1105 BD Amsterdam, The Netherlands; m.j.van.hartingsveldt@hva.nl; 2 Centre of Expertise Urban Vitality, Amsterdam University of Applied Sciences, 1105 BD Amsterdam, The Netherlands; m.stuiver@nki.nl; 3 Department of Public and Occupational Health, Amsterdam UMC Location AMC, University of Amsterdam, 1100 DD Amsterdam, The Netherlands; a.g.deboer@amsterdamumc.nl; 4 Department of Medical Oncology, Netherlands Cancer Institute—Antoni van Leeuwenhoek, 1066 CX Amsterdam, The Netherlandso.husson@nki.nl (O.H.); 5 Societal Participation and Health, Amsterdam Public Health Research Institute, 1105 AZ Amsterdam, The Netherlands; 6 Cancer Center Amsterdam, Cancer Treatment and Quality of Life, 1006 BE Amsterdam, The Netherlands; 7 Independent Researcher, 7522 NB Enschede, The Netherlands; 8 Independent Researcher, 1411 HB Naarden, The Netherlands; 9 Department of Public Health, Erasmus MC Cancer Institute, Erasmus University Medical Center, 3000 CA Rotterdam, The Netherlands; 10 Centre for Quality of Life, Netherlands Cancer Institute—Antoni van Leeuwenhoek, 1066 CX Amsterdam, The Netherlands; 11 Division of Psychosocial Research and Epidemiology, Netherlands Cancer Institute—Antoni van Leeuwenhoek, 1066 CX Amsterdam, The Netherlands; 12 Department Surgical Oncology, Erasmus MC Cancer Institute, Erasmus University Medical Center, 3000 CA Rotterdam, The Netherlands

**Keywords:** adolescents and young adults (AYAs), cancer, higher education, transition to work, qualitative, healthcare, educational and social support

## Abstract

**Background/Objectives**: Adolescents and young adults (AYAs) with cancer often begin their careers later in life and are at risk of negative work-related outcomes. Research into and tailored support programs for AYAs diagnosed during higher education remain limited. An improved understanding of AYAs’ experiences is essential in guiding the development of age-appropriate support programs. This study explored the impact of cancer and the challenges AYAs face in educational participation and the transition to work. **Methods:** A qualitative study was conducted with thirteen AYAs diagnosed with cancer during higher education. Participants were interviewed using a semi-structured guide. In collaboration with patient experts as co-researchers, data were analyzed via thematic analysis. **Results**: Eight analytically derived themes reflected AYA students’ experiences: (1) Meaning and importance of education, (2) Reduced performance, (3) Recovery and expectations, (4) Interruption and delay, (5) Transition to work, (6) Disclosure, (7) Challenges related to the context of students, and (8) Experienced lack of support. The themes were clustered into four overarching thematic categories: Meaningful participation, Impact on performance, Academic progress and career transition, and Challenges in navigation. **Conclusions:** Our findings provide greater insight into the significance of educational participation for AYAs. AYA students encounter challenges stemming from both diagnosis-related changes in functioning and from contextual factors tied to their roles as students and new starters in the labor market. Navigating the healthcare, education, and social systems is complex and AYAs often lack adequate support when resuming their education or transitioning to work. Tailored support programs in healthcare and educational settings should be developed to help AYAs harness their strong motivation to resume studies, enter the labor market, and achieve their full potential.

## Simple Summary

The number of Adolescents and Young Adults (AYAs) living with/after cancer is increasing. Research shows that AYAs often start their careers later, experience negative work-related outcomes, and require occupational support. However, the impact and specific challenges posed by a cancer diagnosis on AYAs enrolled in higher education are poorly understood. This interview study aimed to explore the experiences of AYA students to inform the development of tailored support programs. Our results highlight that education is important for AYAs beyond academic attendance. They also strive to minimize interruptions and delays while coping with reduced performance and age- or context-related challenges, such as financial difficulties or questions around what to disclose about their diagnosis. AYAs experienced a lack of adequate support when navigating healthcare, education, and social systems, suggesting system-level failures and highlighting the need for tailored programs to help AYAs resume their studies and enter the labor market after a cancer diagnosis.

## 1. Introduction

In the previous decade, adolescents and young adults (AYAs; 15–39 years) diagnosed with cancer have been recognized as a distinct group in oncology research and care [1]. Cancer incidence in this group is increasing and AYAs have an overall 5-year relative survival rate of >85% [2]. As a result, an increasing number of AYAs are dealing with the long-term effects of the disease and its treatment, such as an increased risk of chronic medical conditions [3], cognitive impairment [4] and fatigue [5,6,7]. Furthermore, AYAs face specific psychosocial challenges [5,8,9,10,11,12,13,14]. The AYA age range of 15–39 years is developmentally heterogeneous and can be subdivided into four developmental subgroups—adolescents (15–17 years), emerging adults (18–23 years), younger young adults (24–32 years), and older or established adults (33–39 years)—each characterized by distinct core developmental tasks and challenges, and whereby the younger subgroups form their identity, transition towards independence and explore future educational and career paths [15]. AYAs face a long work life, making engagement in both education and work essential from both an individual as well as societal perspective [6,16,17].

However, many AYAs are impeded in their participation in education or the start of their career. Quantitative data from The Netherlands showed that AYAs with cancer are more likely to be unemployed, start their careers at an older age, and face negative financial outcomes for at least 5 years after diagnosis, compared to the control group: adolescents and young adults without cancer [18,19]. In qualitative research, AYAs report that cancer has a negative impact on their study or work plans [5,10,20,21], social connectivity, career growth, financial security [6,13,22,23,24], and overall quality of life (QoL) [17,25].

In their review, Altherr et al. (2023) [26] showed that considerably more research has focused on AYA cancer survivors’ employment outcomes. They emphasized that those diagnosed at a younger age appear particularly vulnerable to adverse outcomes, and highlighted that there is a dynamic interaction between life stages, state of health and the effect on educational attainment, transition to work and financial independence. Younger AYAs are frequently still engaged in education, and although existing studies show that return to education is a significant concern of AYAs [21,26,27,28,29], research with a focus on AYAs diagnosed with cancer while they were enrolled in higher education is scarce. These AYA students face a double transition—from patient to survivor and from student to worker [30]. The existing research suggests that late effects, peer misunderstanding, financial burden, and systemic barriers within academic and municipal institutions significantly affect AYA students’ social outcomes and academic performance [21,26,27,28]. Internationally, educational, healthcare and municipal (support) systems are frequently reported as bureaucratic, inflexible, and difficult to access [21,28,29]. This is concerning, given that often-invisible limitations, such as fatigue and lack of concentration, can create challenges in completing coursework and examinations, both during treatment and in the survivorship phase [21,28,29,31]. Additionally, late effects such as cancer-related cognitive impairment, fatigue, or psychological difficulties can cause stress, as AYAs reported it difficult to explain their often-invisible limitations to peers, their educational institutions and future employers [21,28]. On one hand, non-disclosure can cause distancing between AYAs with cancer and their peers. On the other hand, AYAs also report that disclosure causes feelings of discomfort amongst their peers, changing the way others perceive them. Thus, to avoid a fear of misunderstanding or labelling, AYAs may be hesitant to disclose their cancer status to others [28,32].

While an increasing body of research highlights the negative impact of cancer on education and employment for AYAs [10,16,23,26,28], support programs that address these problems are still in their infancy. Internationally, some initiatives have been launched to develop services to support AYAs participation in education and transition to work. For example, in Denmark and Australia, multidisciplinary support programs address the impact of cancer and its treatment on the academic participation of AYA students [23,33]. They offer AYAs practical support and assist them in planning and adjusting their courses, which are consequently tailored to their individual impairments and abilities. In Denmark [33], a primary contact person is assigned who contacts the AYAs’ educational institution to ensure the best possible conditions for the AYAs to continue and complete their education. The contact person is also tasked with assisting the AYA in navigating local government policies and dealing with financial and legal issues, as suggested by Pedersen and colleagues (2018) [29]. While the topic of education and transition to work for AYAs is on the research agenda, and educational and vocational services seem to be beneficial in keeping AYAs in education and work [26,33], it is currently not clear how AYAs in The Netherlands perceive their own situation. To guide the development of a support program, a better understanding of the impact and specific challenges AYAs face when returning to higher education or transitioning to work after their cancer diagnosis is needed. Therefore, this study aims to explore the experiences of AYAs who are diagnosed with cancer during higher education, focusing on the challenges in their participation in education and transition to work following diagnosis and treatment.

## 2. Materials and Methods

This study is grounded in an interpretivist epistemology, which assumes that reality is socially and subjectively experienced and is best understood by exploring participants’ meanings and interpretations in context [34]. A qualitative study using semi-structured interviews was conducted to gain in-depth insight into the experiences of AYAs diagnosed with cancer while enrolled in higher education, focusing on study participation and their transition to work. Reflexive thematic analysis was used as an interpretive, flexible approach to identify and analyze patterns of meaning within the data. The results are reported in accordance with the Standards for Reporting Qualitative Research (SRQR) [35]. To ensure the scientific rigor of this qualitative study, multiple strategies were used to enhance indicators of trustworthiness, including credibility, transferability, dependability and confirmability [36].

### 2.1. Setting

The setting is relevant to consider the transferability of our findings [36]. We conducted the study in The Netherlands. In The Netherlands, AYAs are typically defined as those diagnosed with cancer between 18 and 39 years of age. Their care is integrated in adult oncology care, whereas patients diagnosed before the age of 18 receive treatment and follow-up care in a specialized pediatric oncology setting. In The Netherlands, around 4000 AYAs are diagnosed each year, including students [37]. Since the Dutch AYA Care Network was established in 2014, specific care for AYAs has been developed and has since been implemented by a growing number of hospitals [38]. However, there is still room for improvement in AYA care with many unmet needs during treatment and adulthood thereafter. Education is one of the topics that is marked as important by the AYA care network [38], but knowledge of how to support AYA students is lacking. Our study is a first step to provide insight into the experiences of AYA students and to develop a support pathway that supports AYA students in their participation in educational and transition to work, thereby improving quality of life.

### 2.2. Study Population and Recruitment

Participants were eligible if they met four criteria: (I) were diagnosed with any type of cancer either within the Dutch binary higher education system—which comprises higher professional education (Hoger Beroepsonderwijs, HBO; universities of applied sciences) and research-oriented higher education (Wetenschappelijk Onderwijs, WO; research universities)—or within secondary Vocational Education and Training (VET, referred to in The Netherlands as middelbaar beroepsonderwijs, MBO) (II) with a first diagnosis between the age of 18 and 25 years, (III) were diagnosed between 2002–2022, and (IV) had sufficient proficiency in the Dutch language to understand and respond to interview questions. Criterion II (diagnosed at 18–25 years) was chosen to ensure that participants were 18 years or older at the time of diagnosis, as those diagnosed before age 18 are treated and followed up in pediatric oncology settings and do not fall within the Dutch AYA age range. Based on the duration of full-time educational degree programmes, a maximum age of 25 years at the time of diagnosis was chosen to predominantly include students for whom studying is the main activity. within this age range. Participants were recruited during the period from 8 February 2022 to 26 April 2022 via the regular digital newsletter and social media channels of the AYA care network in The Netherlands and the Dutch AYA patient advocacy group (SJK). Thirteen respondents met the inclusion criteria and were contacted by the researcher (AB) by phone to receive an explanation of the study, after which they were sent additional written information by email. Prior to the data collection, written informed consent was secured from each participant. Throughout the analysis of the 13 interviews, the research team engaged in iterative discussions and repeatedly reviewed the transcripts, ultimately establishing that no new codes were needed. Consistent with the study’s aim, the dataset was considered to provide sufficient depth and variation to capture the experiences of AYAs diagnosed with cancer during higher education, and recruitment was therefore concluded [39].

### 2.3. Data Collection

The semi-structured interview guide was constructed based on previous scientific literature and findings from critical discussion with OH, MvH and MS. Multiple topics were included in the interview guide. For the current paper, we focused on the specific topics: experiences that participants deemed to be important during their education, impact on performance and participation, support and processes of support, and future perspectives. The draft version of the interview guide was further developed based on empirical knowledge through discussions with a clinical occupational physician who is familiar with AYAs and a representative of SJK (see acknowledgements). This version was pilot tested by AB with an AYA with lived experience of diagnosis during their university studies (FH) and adapted into the final version. The researcher (AB) has a background as an occupational therapist and academic lecturer and has experience in conducting interviews. Awareness of the researcher’s positionality was strengthened through regular discussions of field notes with the supervisor (OH) and a researcher with a background in psychology (MR), fostering ongoing reflexivity. During the interviews, the researcher (AB) used active listening techniques, including paraphrasing, summarizing, and adapted probing questions, to check understanding and enhance the credibility of the data.

Prior to the interview, participants were asked to complete a short case report form (CRF). The CRF included characteristics such as age, sex, and specifics about the type of cancer and treatment. Access to these data was restricted to the first researcher (AB) and OH (daily supervisor), and the data were stored in a secure location separate from the interview data. The interviews were conducted online, using Microsoft Teams (version 1.5.00.10369) by the first author (AB). AB had no prior relationship with the participants, minimizing presupposition and bias in the study process. One week after the interview, the researcher contacted participants by telephone to facilitate the opportunity to reflect or add information. None of the participants wished to modify or add to the information provided during the interview, and they expressed positive reflections on the interview experience.

### 2.4. Data Analysis

The interviews lasted on average 60 min and were digitally recorded and transcribed verbatim. To ensure confidentiality, all identifiers were removed from the transcripts. To explore the experiences of AYAs who were diagnosed with cancer during their university studies—focusing on challenges in their participation in education and transition to work following diagnosis and treatment—we used MaxQDA (version 2022) to facilitate the organization, coding, and retrieval of text segments during the thematic analysis. An inductive, semantic approach was adopted, whereby themes were developed in a data-driven way to reflect the explicit opinions as voiced by the AYAs [40]. In the Discussion, we moved beyond this predominantly semantic approach to offer a more latent interpretation of the themes, reflecting on deeper meaning frameworks such as identity and on the underlying systemic structures. Between 1 February 2022 and 31 October 2022, the interview data were thematically analyzed using the six phases outlined by Braun and Clarke [40,41]: (I) Familiarizing oneself with the data, (II) Generating initial codes, (III) Combining codes into themes, (IV) Reviewing themes, (V) Defining and naming themes, and (VI) Writing the final report. In phase two, two authors (AB and MR) independently coded two transcripts and subsequently compared and discussed their interpretations to enhance researcher reflexivity and deepen the analysis of the codes. In phase III and IV, the themes were defined and discussed during consensus meetings with patient experts, hereafter referred to as AYA co-researchers, who are actively involved as partners in this research (FH, IvV and MB), and OH. Phase five was conducted with the AYA co-researchers (FH and MB) and discussed with the research team (AB, OH, AdB, MS and MvH). Relevant quotes were individually selected by the AYA co-researchers (FH and MB) and AB to illustrate central patterns within the data and were representative of the underlying themes. The agenda for the consensus meetings was sent to the co-researchers by email in advance. At the start of each meeting, the agenda was collaboratively supplemented and priorities were agreed upon, after which the meeting began. During the consensus meetings, the AYA co-researchers were invited to share their interpretations, selected quotes and ideas for theme labels, after which the academic researcher presented their own interpretation, in order to foreground AYA perspectives and limit undue influence of the researcher (AB). In this iterative process, agreement was reached in all cases: the experiential knowledge of the co-researchers was decisive when there was doubt about quotes, whereas the researcher’s expertise was decisive when there was doubt about themes. After agreement was reached on the themes and quotes, these were translated into English by one of the AYA co-researchers, AB and JM. The prolonged involvement of, and discussions with, the AYA co-researchers and the research team enhanced the researcher’s reflexivity. It also strengthened the credibility and confirmability of the findings, as peer debriefing allowed for critical examination of emerging interpretations and potential biases [36]. In the last phase, writing the report, we intentionally stayed close to participants’ own words and discussed the language used with the AYA co-researchers with lived experience. The active role of AYA co-researchers in the process of analysis and selecting illustrative quotes enhanced the credibility and confirmability of the findings [36]. Throughout the process, we maintained a reflexive approach and kept analytic memos to ensure transparency regarding the research process and the rationale for our decisions and enhance dependability [36].

### 2.5. Ethical Considerations

The study was approved by the Institutional Review Board of The Netherlands Cancer Institute, which declared that the Medical Research Involving Human Subjects Act (WMO) does not apply to this study (IRBd21-294). All respondents provided written informed consent, and their identities were protected using project ID numbers. To ensure privacy, age and clinical data were not combined in “Table 1. Characteristics per respondent”.

## 3. Results

### 3.1. Sample Characteristics

Thirteen AYAs were interviewed. They were enrolled in either a bachelor’s program at a university of applied science (HBO; n = 4), or a bachelor’s (n = 5) or master’s (n = 4) program at a research university (WO). Respondents had a mean age at diagnosis of 21.3 years (range: 19–25), and 10 were female (cisgender). Further respondent characteristics of the study population are shown in “Table 1. Characteristics of study population”. Respondents were assigned a number (R01–R13) to identify quotes from different respondents throughout the report.

### 3.2. Thematic Categories and Themes

**Figure 1:**
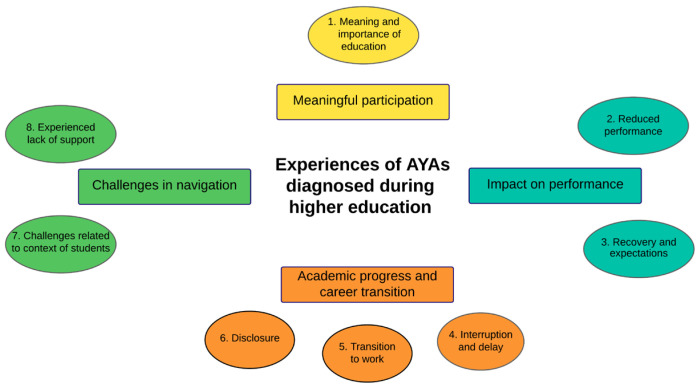
Visualization of thematic categories and themes.

**Figure 2:**
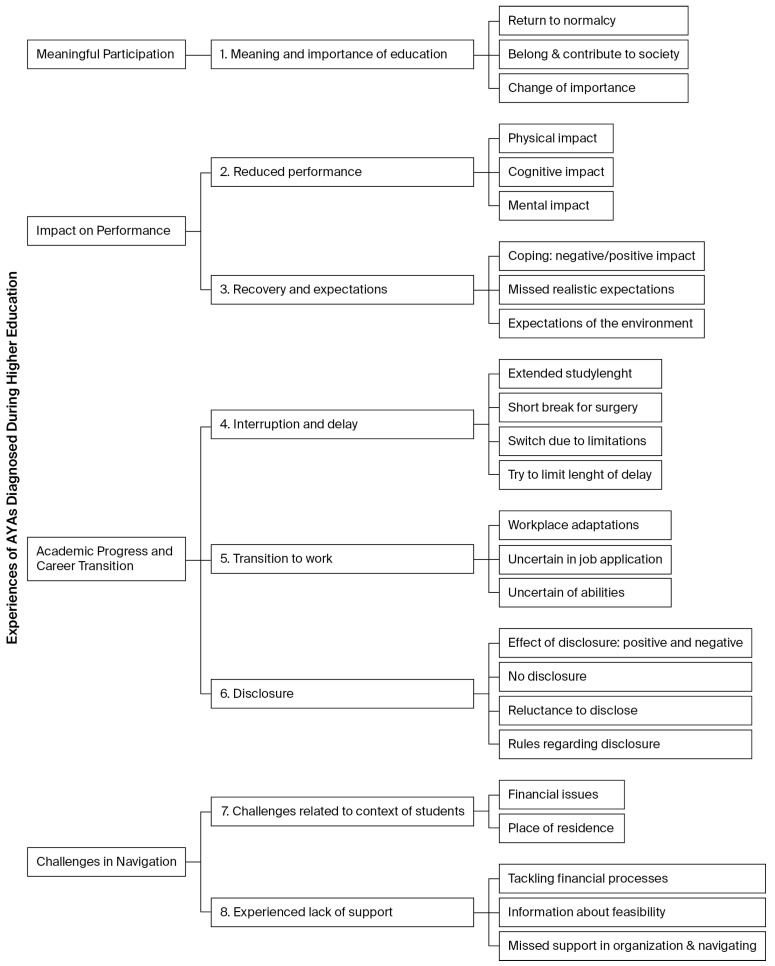
Thematic categories, themes and subthemes of AYAs diagnosed during higher education.

We identified 8 themes in the experiences and challenges faced by the AYAs, which we clustered into four overarching thematic categories to support a clearer interpretation of the findings: (1) Meaningful participation, (2) Impact on performance, (3) Academic progress and career transition and (4) Challenges in navigation. This is visualized in ‘Figure 1. Visualization of thematic categories and themes’. The subthemes identified within each of the eight themes are shown in ‘Figure 2. Thematic categories, themes and subthemes of AYAs diagnosed during higher education’, and are further described and illustrated with quotes in the section below. In the text, thematic categories are the headings (bold and italic), themes are shown in bold, and subthemes are underlined in the text, provided with illustrative quotes.

#### 3.2.1. Meaningful Participation

**(1)**  **Meaning and importance of education**

Return to normalcy; belong & contribute to society

AYAs felt that engagement in education gave them a sense of “normalcy.” Continuation of their education provided structure in their lives, gave them hope, limited their time to think about their illness, and social contact with their peers created a distraction from illness. Their education offered them a role beyond that of a patient—creating a sense of belonging—and enabled them to contribute meaningfully to society.

*“It also gave me a lot of structure, support, and strength. It made me think ‘Yes, I’m just studying’.”*

*(R11)*

*“You’re in a world surrounded by people who are studying, so you also want to be engaged in it.”*

*(R7)*

Change of importance

Prior to their diagnosis, education was of great importance to all AYAs. However, respondents reported a shift in perspective after their diagnosis, with some prioritizing other aspects of life instead of their studies. Other AYAs found their studies to be even more important. They saw resuming their studies as a way to integrate into society and re-establish a sense of belonging. Some realized that their diagnosis did not change their perspective on their education.

*“And during my illness, I became even more aware of how important other things are besides studying.”*

*(R5)*

*“And that was the idea—to keep my studies going during treatment. If I study, if I stay enrolled, then I’m not sick. So yes, I still think my studies were actually more important than my own health.”*

*(R8)*

#### 3.2.2. Impact on Performance

**(2)**  **Reduced performance**

Physical impact

AYAs reported fatigue as a symptom before diagnosis and primarily because of treatment. Fatigue hindered their participation as they required more sleep, continued to feel fatigued after many hours of resting, and experienced reduced or limited capacity in social activities, studies and work. Limitations in energy caused dilemmas, as combining recovering and studying proved too demanding. Other physical limitations, such as stomach problems, limited traveling and participation in activities outside the home. Plus, the fine motor skills required for their studies were negatively impacted by neuropathy/tremors. Further concern was raised by AYAs as to how this might affect their future desired professions.

*“I experienced a level of fatigue that’s just impossible to explain [...]. ‘I’m so tired, I’m so, so tired.’ Even if I slept nine or ten hours a night, I’d wake up feeling as if I had only slept for ten minutes.”*

*(R4)*

*“I still have issues with my fingertips. Of course, I would have preferred not to have it [neuropathy]. But oh well, I got it for free.”*

*(R12)*

Cognitive impact

Disrupted cognitive functioning was a primary reason that AYAs were limited in participating in education. They reported difficulties with concentration and information processing, which impeded their ability to study.

*“The focus wasn’t there, the concentration wasn’t there, it just didn’t register. You feel like there’s some kind of cloud over your brain. You’re sitting there, but what’s being said? No idea.”*

*(R8)*

Mental impact

Cognitive and physical impairments severely affected AYAs’ daily functioning and their concerns regarding their capabilities negatively affected their mental health. AYAs reported debilitating panic attacks, anxiety, and a fear of having a “chemo brain” (e.g., problems with memory, attention, processing speed, and executive functioning after chemotherapy). They reported reduced confidence in their capabilities and a fear of being unable to meet the expectations of future employers, which affected their confidence in job applications.

*“But really, as soon as the chemo was over, I suddenly thought: ‘Wow, I’ve actually lost who I used to be. I’ve lost my independence, my ability to think clearly is gone.’ I had panic attacks because I was too afraid to go outside alone.”*

*(R8)*

AYAs experienced periods of emotional imbalance, for example, due to medication or stress induced by annual check-ups. Cancer-related changes in appearance also negatively impacted participation in education and led to the postponement of job applications. Short hair, baldness, or not having the stereotypical appearance of a cancer patient (e.g., treatment-related hair loss), evoked a need to justify or explain themselves to others. For another participant, the diagnosis changed their existential perspective.

*“I always had long hair, like I do now. But back then, I didn’t—I had black curls. And you just don’t feel like yourself. Yet, that’s the introduction you make into this new world you’re stepping into.”*

*(R5)*

**(3)**  **Recovery and expectations**

Coping: negative/positive impact

AYAs emphasized that they wanted to get back on course with their university studies as soon as possible and used coping strategies to keep moving forward. Retrospectively, AYAs mentioned that they could have been kinder to themselves, having previously felt the need to feign resilience. Were they to relive the experience, AYAs stated that they would take more time to recover and restart their studies less intensively. AYAs reflected on the negative impact of their coping strategies: asking too much of themselves resulted in burnout, feelings of failure, or not taking sufficient time to process their experience.

*“With what I know now, I would have never started a full-time study program, only to be completely burned out within a month.”*

*(R6)*

Some AYAs described pushing themselves too hard as a typical trait of their age. Although the majority mentioned the negative consequences of their choices to resume education early after diagnosis or treatment, some AYAs also highlighted the positive effects of this coping style. They explained it was helpful, as it distracted them from their illness and enabled them to partially continue with their normal life.

*“That it really helped me a lot to, yes, continue my studies. And yes, actually, on the one hand, it’s just a huge distraction; it makes your normal life continue a bit, despite everything.”*

*(R11)*

Missed realistic expectations

Some AYAs expected to continue their studies during treatment or planned to resume them shortly thereafter. However, many respondents reported that the treatments’ effects were significantly more intense than expected and limited their ability to study. AYAs reported that unclear expectations led to overexertion and had a negative impact on their wellbeing. It caused frustration and feelings of failure and disappointment at the prospect of having to postpone their studies again after resumption. Retrospectively, AYAs reported they demanded too much of themselves, subsequently regretting not taking the time to recover or process the impact of the diagnosis.

*“You notice that concentration is sometimes difficult, keeping up with lectures is a challenge. You’re still in the recovery process. You expect to immediately return to your old level, but that’s just not how it works.”*

*(R7)*

*“I had such a bad experience—I had to quit after a month, and it really made me afraid of studying again.”*

*(R6)*

*“The frustration was psychological. I wanted to move forward, but I was using the wrong frame of reference. What did it cost me? My enjoyment of studying, I think.”*

*(R12)*

Furthermore, AYAs’ unclear expectations led to not AYAs not requesting support when it was needed. One AYA explained that they did not ask for support because their diagnosis had been known for a more than a year. According to their frame of reference, it was no longer appropriate to seek help, as they believed their time of need had passed. Moreover, the lack of realistic expectations led to unfavorable practical situations such as continuing to rent their student apartment with the expectation of being able to resume their studies shortly after chemotherapy.

*“I kept my room, but I was a bit naïve. I thought I’d be able to move back there within six months. But a month ago [note: 1 year after diagnosis], I cancelled the lease.”*

*(R1)*

Expectations of the environment

Expectations from peers and family created additional pressure on AYAs, who subsequently felt the need to perform at their maximum level or beyond. As one AYA explained, people in their social environment often assumed that AYAs would be able to perform at the same level after chemotherapy as they did before diagnosis, failing to recognise the lasting impacts of treatment.

*“For a lot of people, chemo is the cancer treatment, and once you’re done with chemo, you’re cured, you’re finished. For the people around you, you’re supposed to be better. But that’s not how it works.”*

*(R8)*

#### 3.2.3. Academic Progress and Career Transition

**(4)**  **Interruption and delay**

Extended study length, a short break for surgery or switch due to limitations

Almost all respondents extended their education enrolment duration due to treatment, aftercare and recovery. Most AYAs interrupted their education immediately due to treatment. Some tried to continue at a slower pace and paused their education when treatment became more intensive, with one respondent taking just a two-week break for surgery. After recovery, two respondents changed their course, realizing that their current educational program or professional aspirations were incompatible with their post-diagnosis capabilities and therefore had to be changed.

*“When I got the diagnosis, I immediately stopped studying. [...] Cancer became such a priority that everything else just fell away. I didn’t even think twice about it. I think that’s very normal, very understandable.”*

*(R6)*

*“And then I thought, well I’ll have to do it part-time. That ruled out medicine because you can’t do that part-time. So, psychology it was.”*

*(R6)*

Try to limit the length of delay

Respondents aimed to limit delays to their education for several reasons, such as to try to live up to high expectations from themselves or their parent(s), limiting the financial impact, maintaining the meaning provided by being engaged in their education, maintaining contact with peers, or to mitigate feelings of failure.

*“If I stop now and have to start again in a year, that would be awful. I’d fall so far behind my peers, while right now those are the people who can help pull me through.”*

*(R11)*

**(5)**  **Transition to work**

Workplace adaptations

In reflecting on future work prospects, AYAs expected that they would require post-cancer workplace adaptations, such as working fewer hours due to their illness or adjusting their planned career path.

*“I think I could work ten to fifteen hours a week, which would be my goal, while also being able to go to the gym, maintain a social life, and keep up with household chores.”*

*(R8)*

Uncertain of abilities and in job applications

AYAs were uncertain about their capabilities post-cancer. They were concerned about their energy levels, and their subsequent ability to work regular hours post-treatment. Furthermore, AYAs feared developing “chemo brain” or limited cognitive abilities. They also faced uncertainty in future job applications. Some were afraid of being judged or of being unable to meet the requirements, such as the required number of working hours. Others questioned how to frame their experiences as a survivor of cancer positively, as further described in subtheme 6 “Disclosure”.

*“In the beginning, I was really afraid that I was suffering from chemo brain […] So for me, it also brought some uncertainty, like: yeah, what am I actually still capable of?”*

*(R11)*

*“Sometimes I worry—can I even work a full day? And how will employers view that?”*

*(R6)*

*“How do I incorporate my cancer experience into my CV and the image I present of myself? So that, in a way, it empowers me.”*

*(R11)*

**(6)**  **Disclosure**

Rules regarding disclosure, reluctance and non-disclosure

Issues around disclosure were frequently mentioned. Rules regarding disclosure to future employers were not clear and AYAs were reluctant to disclose their diagnosis. Some did not disclose their diagnosis due to a fear of being treated differently, underestimated, or overprotected. Others did not disclose their diagnosis because they changed workplaces, or the supervisor was new in their team. Some respondents did not disclose their diagnosis due to the belief that workplace adjustment was not possible, and some AYAs assumed it would no longer be relevant given the time elapsed since diagnosis.

*“But I think it’s good to know what you’re obligated to disclose and what you’re not. There used to be a rule that you had to tell them, unless it had been five or ten years. I remember hearing something like that.”*

*(R1)*

*“But look, I have two years of work experience now, but I still wonder: should I mention it again, or should I not?”*

*(R2)*

Effect of disclosure: positive and negative

AYAs who disclosed their diagnosis primarily reported positive experiences, such as receiving support, being able to “be themselves”, lowering the expectations of others (in line with AYAs capabilities), and colleagues understanding their needs during hospital check-ups or periods with reduced employee performance. A few AYAs had negative experiences due to a lack of understanding from their teachers or faced job rejections after disclosure.

*“Cancer is simply a part of me. If I didn’t talk about it, it would almost feel like denying a part of myself. So, I really wanted to share it, also because it has had such a huge impact on who I am and how I approach life.”*

*(R11)*

*“I choose not to bring it up to colleagues, because, as I mentioned earlier, I want them to see me for who I am and not treat me differently because of it.”*

*(R3)*

#### 3.2.4. Challenges in Navigation

**(7)**  **Challenges related to context of students**

Place of residence

For AYAs who lived alone in their university city, the implications of their diagnosis resulted in residential changes; AYAs moved back home with their parents after diagnosis and back to their university city in a later phase of reintegration. Although living with their parents was necessary during treatment and recovery, it reduced the academic options available to them. Firstly, moving in with parents post-diagnosis often increased the physical distance between AYAs and the university. This impeded part-time participation due to the energy costs of travel. Furthermore, AYAs who lived with their parents and tried to study part-time experienced changes in their study routines. They were unable to study together with peers and lacked peer support. In later phases of reintegration, AYAs moved back to their former residence in their university city. In this phase, their place of residence once again caused related challenges, such as limitations in accessing the care and support programs provided in treating hospitals closer to their parental address.

*“In principle, you can do any course through self-study, but it’s really difficult when you haven’t studied for a year. You’re used to studying with peers, so when you’re at home, you’re completely alone.”*

*(R1)*

*“But then I called general practitioners in [location of university], even though my official general practitioner (GP) was still registered with my parents. I asked: ‘Can you recommend a psychologist who also understands cancer and can guide me through this?’ I called two or three different GPs, and they all told me: ‘No, that is not what we are here for. You would have to register with us first before we can help you.”*

*(R8)*

Financial issues

The financial aspects of a diagnosis directly impacted the choices that AYAs made regarding their studies. AYAs experienced limited financial support from the university, municipality, or government after their diagnosis. Those who applied for financial support encountered strict limitations, such as receiving only partial support based on their level of study or outright denial if their situation did not meet the specified criteria.

*“I think I claimed a small part or something. I don’t remember, but not full, because I hadn’t gotten cancer in my undergraduate year, but in my master’s year. So that was kind of such a crooked arrangement. They still said, if you had had that a year earlier, we could help you, but not now. So that was another thing that I thought, what a crooked system.”*

*(R2)*

Many AYAs became financially dependent on parents and/or a partner, which was perceived as a burden and conflicted with the development of independence. Students who, due to their part-time employment, were entitled to employer-provided sick pay during periods of illness had fewer concerns.

*“If you don’t have financial support, you become dependent on your mom and your partner, which is the last thing you want. That was a big reason why I wanted to graduate as soon as possible and have my own income. Because when you’re 25 or 26, you really don’t want to be financially dependent—not on your parents, and not on your partner. You just want to do your own thing.”*

*(R11)*

AYAs felt that the lack of additional funding (beyond familial) led to unfavorable situations. One AYA stated that they felt a dilemma between stopping their studies to qualify for social welfare or resuming their studies part-time while incurring extra costs. Another AYA described this as being trapped: the municipality did not provide support, but advised them to take out an additional loan, even though this would impact their ability to qualify for a mortgage at a later stage. For several AYAs, financial issues were the main motivator to start studying. For example, university enrolment enabled them to receive a state education grand again.

*“That was also one of the reasons I went back to studying—so I could get state education grand again and live on my own again.”*

*(R12)*

*“I think these are specific challenges for AYAs. You’re in a phase where nothing is settled, yet you don’t have a permanent contract, a steady income, or a home. And then, because of the illness, you’re suddenly stuck.”*

*(R11)*

AYAs who changed courses due to disability had their student loans forgiven and started a new program with renewed rights. Renewed rights facilitated a return to studies, but the process to access them was cumbersome.

*“We also applied for new student loan entitlements, so then you’re basically saying that I would never be able to finish medical school because of my situation now, that that means I get to start all over again.”*

*(R6)*

*“Yes, I had to go specifically to a doctor in [city, not in the neighborhood] for that, which I thought was a little crazy, but okay. And I think that was just a general practitioner there. And I had to go there… I had looked up on the Internet what kind of forms they have to have and have to sign. […]. And then the right box was checked and I could get my money back.”*

*(R5)*

**(8)**  **Experienced lack of support**

Missed support in organization & navigating

AYAs mentioned that staying connected with or reintegrating into their study required a lot of energy, and they faced extensive bureaucratic challenges. Throughout this navigation process, they lacked practical and psychosocial support, while illness and recovery were already all-consuming. The absence of guidance and information from their university or hospital about available support contributed to feelings of loneliness and exclusion.

*“You had to figure everything out yourself, and I just didn’t have the energy for that.”*

*(R2)*

*“I didn’t know anyone around me, I had no idea where to go. You just feel incredibly lonely and think I’m never getting out of this.”*

*(R4)*

*“You need support during treatment, but especially afterwards when you’re trying to pick up your studies again. That’s when you really run into challenges, and support in that phase is so important.”*

*(R9)*

Information about feasibility

For the majority of AYAs, it was difficult to decide when to return to education, with many realizing in hindsight that they had unrealistic expectations of their capabilities after treatment. AYAs elaborated on the information they were given during their trajectory by hospital professionals. In most cases, professionals did not discuss how AYAs’ adjusted abilities and recovery align with their academic program. In situations where it was discussed, AYAs experiences differed. For one, it was helpful to know what to expect, as it fostered a sense of acknowledgment and tranquility, improved faith in recovery, and increased their understanding of when to seek support. Others were informed about the impact of treatment on their abilities, but had negative experiences, as the impact was more severe than outlined in the hospital. This resulted in feelings of frustration or caused AYAs to consider themselves “stubborn.” Thus, they were informed but did not expect that the impact outlined by the professional(s) would be that severe for them personally, and they had to confront their own feelings and thoughts in relation to this. In a few cases, their parents or the student counsellor noticed that the AYAs were overwhelmed and helped them to decide when to interrupt their studies.

*“But professionally, really no one [addressed the expectations of recovery]. Not even the doctor. Yes, he said: ‘Yes, you can...’ Yes, I don’t want to say nonchalantly, the way he told it, but he was like: ‘Well, people do get tired after the treatments, but generally they come out fit again. So that was kind of the hope that was given, that it should work out again in September.”*

*(R12)*

*“My, oh yes, my doctor who had said [...], ‘You can study or work alongside this illness.’ And then I thought, ‘Oh, if anyone can do that, I can’. So, I did my radiation therapy, I did it in [city], because I thought, ‘I can study in addition to that.’ However, I wasn’t able to do anything about my studies during radiation, nothing at all.”*

*(R8)*

*“But in terms of focusing on the study, it was said that the condition and concentration would go down. I also knew about myself: I’m younger than the average person who goes through this. So, you hold onto the vain hope of being able to do something and stay busy during your sickbed. But I found out very quickly that that was not the case.”*

*(R7)*

Tackling financial processes

AYAs highlighted the complicated process of obtaining additional benefits, noting both difficulties in understanding their rights and the lack of professional guidance. This required independent initiation and management of bureaucratic processes.

*“I’m not entitled to anything. I completely fall through the cracks. I’m not eligible for benefits or anything, and I’ve fought hard to find out if I was. But no, I’m not.”*

*(R4)*

## 4. Discussion

This qualitative research is the first conducted in The Netherlands to explore the experiences and challenges faced by AYAs receiving a cancer diagnosis during their university studies. Through in-depth interviews and in collaboration with AYA co-researchers as patient experts, we gained insight into the critical importance of educational continuity for AYAs, as well as the challenges they face when diagnosed with cancer during their studies.

The exploratory design of this study enabled the collection of rich, in-depth data that adds to the currently limited knowledge of the experiences of AYAs in higher education. Our findings contribute to a deeper understanding of their experiences by offering new insights into the meaning and importance of education and its intersection with personal and contextual factors in the attempts of AYA students to participate in education. The data also suggest potential underlying mechanisms and the role of systemic structures in reintegration. Ultimately, these insights could direct future research and drive program development. Across the eight themes, four overarching thematic categories emerged: (1) the meaning of study participation, (2) the impact of diagnosis and treatment on AYAs’ ability to study, (3) the impact on academic progress, and (4) their challenges in navigating the diverse systems. Consequently, our findings highlight the importance of educational participation and the personal and contextual constraints that limit it. Hence, insights gained from this research can support the design of age-appropriate support strategies for AYA students.

*(1)*  *The meaning of study participation: beyond academic attendance*

The interviews indicate that, although the experiences of AYA students differ, they share a motivation to resume their studies that extends beyond academic attendance alone. Studying provides AYAs with belonging, meaning, and a sense of contribution. This form of participation not only gives AYAs a role but also provides a sense of societal contribution. These social processes are important in the psychological process of *identity* development that is essential to the AYA stage of life [15,42].

Earlier research, with a focus on cancer and employment, indicated that cancer survivorship might lead to the re-evaluation of personal priorities and changes in one’s identity [27,28]. Our findings suggest that these processes similarly manifest among AYAs in educational settings. Several respondents described how their diagnosis or cancer-related limitations altered their perception of the importance of their studies, occasionally resulting in AYAs adjusting their future plans. Furthermore, interviewees felt that their self-identity was challenged due to changes in their appearance—such as chemotherapy-induced hair loss—which caused insecurity. Experiencing a sense of “falling behind”, social disconnection from peers, or insecurity about their body image can place AYA cancer survivors at increased risk of reduced self-esteem and a disrupted sense of identity, as shown in earlier research [28,31,43]. Thus, our results suggest that the significance and meaning of participating in education are intertwined with age-related identity formation. This builds upon previous work, which, from a life-course perspective, highlights how serious illness can reshape identity formation [15,42], and indicates that age-appropriate support in which these processes are recognized and explored is important for AYAs with cancer. Furthermore, Fardell et al. (2018) [16] reported that the interplay of interruptions to AYAs’ education, their career path, and a loss of financial autonomy can reduce AYAs’ sense of identity. In the interviews, AYAs elaborated on their desire to regain their student roles, which they described as not only essential in regaining financial autonomy, but also in regaining social *independence*. AYAs stated that this could be achieved by living independently in the city of their university, rather than with their parents. The process of becoming financially independent is an important value for AYAs, as described previously [16,21,27,28]. Our findings indicate that, for AYAs, education functions not only as a route to *financial* independence but also as a means to reclaim *autonomy* in a broader sense.

Furthermore, education can function as a *coping mechanism* for AYAs—an anchor that provides structure, purpose, and a route to ‘normalcy’. Although their motivations were unique, all respondents stated that they resumed their education as soon as they were able to. The drive to resume their normal lives as soon as possible has been described as an advantage in earlier research [16,17,28,29,44], but our findings also reveal downsides of this coping style. AYAs often overextend themselves by taking on workloads or responsibilities that exceed their physical or cognitive capacity, resulting in negative health outcomes such as burnout, anxiety, and decreased self-esteem. Pederson and colleagues [29] described “the drive of youth” from the perspective of professionals working in hospitals, educational institutions and social services. Professionals state that this drive can be attributed as advantageous, but can also be detrimental as AYAs, as they tend to overexert themselves by taking on more than they can handle [29]. Our findings confirm this from the perspective of AYAs themselves.

Overall, the emergence of this theme highlights the need to recognize and acknowledge age-specific processes—such as identity development and emerging independence—in resuming education, and to address these with individualized, age-appropriate support.

*(2)*  *Impact on performance: reduced capacity and shifting expectations*

The previously described impact of cancer and its treatment on the daily physical and cognitive functioning of AYAs is well-known [6,16,21,26,27,44] and is further supported by our research. Although international literature emphasizes that it is important to provide age-specific information about late effects [21,26], our findings suggest that in practice, AYAs often felt unprepared for the impacts of a diagnosis and subsequent treatment on their university studies or transition to work. This led to practical challenges and distress. To select a level of engagement in education that aligns with their abilities, AYAs should be made aware of the impact and late effects of cancer and its treatment. Davis and colleagues [44] underlined the importance of providing information on cancer symptoms, side effects and late effects to enhance knowledge, skills and self-efficacy in self-management. Our research shows that, even for students who are recently diagnosed, suitable information was often not provided. The provision of adequate information about altered capacities seems to be an essential step in enabling AYAs to manage the demands of their studies, and healthcare professionals involved in AYA care should recognize their role in discussing this with AYAs. We recommend that this task be clearly embedded in clinical pathways and assigned to a specific healthcare professional to ensure that it is systematically addressed. Moreover, our results showed that—even when AYAs received information about their potentially altered capacities—this did not always enable AYAs to develop a manageable study program. One explanation might be that AYAs tend to underestimate the impact of diagnosis and treatment on their academic performance [21,27]. Alternatively, another explanation could be related to the content, delivery, or timing of information provision. The verbal manner in which information is often communicated might not be appropriate. Further, the timing might not be optimal or of adequate frequency, and the role of digital information is unknown altogether. In summary, AYAs face physical, cognitive, and psychological challenges that impede their educational participation, and they are often not or inadequately informed about late effects or strategies for managing these alongside their studies. Recognizing their strong motivation while clearly informing them about the potential risks of returning to education too soon may improve their long-term academic outcomes and overall satisfaction with their educational path.

*(3)*  *Academic progress and career transition: emerging uncertainties*

Interruption, delay, and uncertainty were central experiences across all respondents, and are in line with previous findings in Europe [21,27,28]. Our results illuminate the specific contextual factors that place AYA students in a vulnerable position and challenge their academic progress. Firstly, a key characteristic of education is that it continuously presents new knowledge and skills to be acquired, a dynamic mirroring that of starting a first/new job. Our results highlight that this instability requires AYAs to apply skills and knowledge without relying on familiar routines, evoking feelings of uncertainty about their abilities. Secondly, students frequently encounter situations in which their social environment changes. Delays in their education may necessitate joining a new class, and internships, individualized courses, or a first job involve interacting with new peers or colleagues. These circumstances can create concerns around disclosure, such as fear of stigmatization, as described in “3.2.3 Academic Progress and Career Transition (6) Disclosure.” Uncertainties in how to communicate with employers or peers about the effects of the disease and its impacts on career development and employability are well-documented [21,27,32,45]. Our findings support the conclusions described by Rabin (2020) in that there should be a supportive and inclusive environment that facilitates disclosure for AYAs who wish to do so [32]. Our results support the presence of these uncertainties and might be particularly evoked by the instability of their social environment. This includes meeting new classmates or beginning their first jobs, further amplifying uncertainty around disclosure. Thirdly, while AYAs may delay/interrupt their education, their peers continue on a typical timescale. Due to standardized educational pathways, AYAs may re-enter education without familiar peers, thereby lacking peer support during their reintegration into a new class. This negatively impacts their sense of belonging, well-being and reintegration, as described previously [21]. The rigidity of academic/systemic structures had negative consequences for some AYAs, such as around course durations/deadlines, assessment schedules, and access to accommodation. Together, these factors contribute to the debate that academic delay reflects a mismatch between institutional structures and the unpredictable trajectory and late effects of cancer treatment, rather than individual failure [21]. Lastly, the transition to work introduces additional challenges, including concerns about employability and uncertainties as to whether previous aspirations remain feasible. This is particularly challenging in the absence of prior work experiences to draw upon. These contextual factors are intertwined with the experiences of AYAs who face delays in their educational trajectory or who enter the workforce following a diagnosis during their studies. This situation renders them particularly vulnerable, highlighting the importance of professional awareness and support.

*(4)*  *Challenges in navigation: disclosure, contextual barriers, and perceived lack of support*

An additional key finding from our research is the arbitrariness of the available support for AYA students. AYAs provided insight into the complexity of educational, healthcare and social systems in The Netherlands, which were frustrating to navigate, especially when seeking financial support. These findings corroborate previous research in other countries [16,27,29,46]. Although the Dutch educational system aims to be inclusive and accessible to all students [47], our findings indicate that AYAs experience barriers in access to education and financial support. Higher education institutions in The Netherlands are legally required to promote accessibility and provide reasonable accommodations for students with disabilities, and each institution has considerable autonomy in how these rights are implemented and communicated [48]. As a result, support arrangements are highly variable and often difficult for AYAs to identify and navigate. Furthermore, in The Netherlands, there is no structural collaboration between healthcare and educational institutions. Our findings indicate that AYAs shouldered the burden of navigating administrative systems themselves. Some were able to navigate the systems effectively, but at the cost of their already limited energy. Others reported a lack of the energy required, and some were not provided with adequate information to co-ordinate their reintegration independently, causing social inequalities and adverse educational outcomes. In 2018, Büscher-Touwen and colleagues [49] described several organizations in The Netherlands that can support students with a disability, but both collaboration between these parties and a sense of responsibility are lacking. However, while their study focused on the gap between education and the transition to work, our study indicates that this lack of ownership and collaboration persists, and that students also recognize this gap between the hospital and their educational institutes. Guidelines on how to support the reintegration of AYA students are still lacking for both hospitals and educational institutions, and AYA students do not feel adequately supported. Individual, cancer-related and age-related challenges regarding returning to education interact with the systemic structures in which they are embedded. How challenges manifest, and how substantial these problems are, is closely intertwined with country-specific systemic structures, which should be taken into account when interpreting the findings. The current healthcare and educational system in The Netherlands may therefore cause inequalities in access to accommodations in education, care/reintegration programmes, and financial support. Our findings indicate that AYAs should be supported in navigating the complexities of the system by professionals, namely healthcare professionals (HCPs) and educational professionals (EPs), who are adequately equipped to provide the necessary information. Such an initiative has recently been developed in Denmark [33] and was also suggested in a recent review by Altherr and colleagues (2023) [26].

Beyond the lack of professional ownership for supporting AYAs in their educational experiences and transition to work, it is also pertinent to discuss AYAs self-management expectations. In education, and Dutch society more broadly, there is an ongoing shift towards a participatory society [47] in which people are expected to take control of situations themselves and live in a self-directed manner. Our results show that, although self-management is age-appropriate, not all AYAs are able to adopt this role. Energy limitations, limited self-awareness in ability and insufficient knowledge about reintegration—including legislation, financial considerations, and available support—often hinder AYAs’ ability to manage their return to education and transition to work. While the literature has predominantly conceptualized disclosure as an individual and psycho-social issue rather than as a legal and institutional one, our findings suggest that the absence or ambiguity of institutional disclosure requirements may contribute to uncertainty and undermine self-management [32,50]. Future research and support programs should examine how legal and institutional frameworks shape AYAs’ self-management abilities, and encourage a proactive approach in which professionals actively guide AYAs through planning, preparing, and navigating their educational and vocational pathways.

Our findings reveal that, for AYAs in higher education, studying is not solely aimed at preparing for a future profession. Their studies also serve as a setting for engagement in social integration, in developing a sense of belonging, and in contributing to their journey towards (financial) independence. These are the hallmark developmental tasks during this specific phase of life. While their motivation to quickly resume studies is an advantage, AYAs often face difficulties in performance and in managing their capabilities to meet academic demands. Existing information and support do not always result in manageable educational programs. Additionally, the context in which students find themselves places them in a particularly vulnerable position. Educational delays and changes in classes are characterized by a constantly changing environment, reintegration without familiar peers, and other social challenges. This vulnerability is further compounded by changes in residence, financial insecurity, and relatively unstable career positions. Moreover, the complexity of navigating healthcare, education, and social systems can hinder study participation and the process of resuming or continuing education.

This work underscores the need for age-appropriate and context-specific knowledge to support AYAs in their educational and vocational transitions following a cancer diagnosis. They also highlight the importance of advocating for personalized care aligned with AYAs’ needs, while critically addressing systemic structures that may impede their development.

### 4.1. Strengths and Limitations

This exploratory study aimed to gather detailed insight into the experiences of students living with or after cancer, serving as a precursor for future research. The findings should be interpreted in conjunction with the study’s strengths and limitations. Rather than suggesting generalizability, the design and scope of this study indicate that the findings should be understood within the specific characteristics and context of the recruited population—AYAs in higher education in The Netherlands—and are not directly generalizable to the wider population. Another characteristic of our study design is that it offers a cross-sectional snapshot of the experiences of AYAs at a single point in time. Future longitudinal research, following AYAs over time, could strengthen our findings by tracing how experiences evolve in different survivorship stages.

We conducted and analyzed 13 interviews, after which we considered the in-depth information sufficient to identify recurring patterns in the AYA student experience. Our findings should be interpreted with the reached target group in mind. We interviewed 13 AYAs, and although there was an equal distribution of study years in which the respondent was diagnosed, all respondents were enrolled in higher education, either at universities of applied sciences (HBO) or at research universities (WO). While the invitation to participate in our study was not limited to students enrolled in higher education, only these students responded, whereas no Secondary Vocational Education and Training (VET, MBO) students came forward. Given the exploratory nature of our study, we decided not to undertake additional targeted efforts to recruit VET students, but instead to narrow our scope. This strengthens the in-depth insights in experiences of students from higher education, but limits transferability to the experiences of VET students. Future research should assess whether the findings hold in a broader population, including participants who are enrolled in VET. Another limitation in our sample is that our participants predominantly consisted of females (77%). This limitation is commonly observed in qualitative AYA cancer research, in which males are less likely to participate [51,52]. In a recent review, Altherr et al. (2023) describe female gender as a determinant of adverse educational outcomes [26]. Our results indicate likewise. For example, male respondents in our research experienced less burden due to less intensive treatment, or stated that their drive to return as soon as possible was predominantly positive, without negative consequences afterwards. As disclosure was one of the themes, the gender imbalance in our study population may also have influenced the themes that were identified. Therefore, the findings of this exploratory study should be quantified across a larger and gender-balanced sample. Another limitation that may have impacted our findings concerns the recruitment strategy. Recruitment via the AYA care network and a patient advocacy group (SJK) through social media may have preferentially included AYAs already connected to support networks and willing to share their experiences publicly, thereby potentially underrepresenting those less inclined to public disclosure or facing greater social difficulties. Although online and social media invitations are effective for reaching AYA respondents, for future studies, researchers should consider adding more conventional approaches, such as hospital-based outreach, mail, or phone calls, which might better reach more marginalized students [52]. The use of interviews provided valuable in-depth data; however, the possibility of recall bias should be considered when interpreting the results. Although positive, neutral and negative experiences were shared, the potential overrepresentation of salient experiences should be kept in mind when interpreting our findings, as emotionally charged experiences are more easily remembered than neutral ones. In our sample, all participants had been diagnosed after 2012, even though the inclusion window formally covered 2002–2022. We decided not to undertake additional recruitment to include AYAs with a diagnosis before 2012, as our data were rich enough to describe their experiences and a more recent diagnosis was expected to better mirror current care practices and social policy. Most respondents had recent experience and were still enrolled in education, but for 3 respondents, the time since diagnosis was greater than 5 years and they were all working. We acknowledge that a timespan of 10 years might have influenced these participants’ experiences. It may also to some extent impact the transferability of the findings, as improvements in treatment might currently cause fewer side effects or long-term burden impacting educational participation, compared to a decade ago. Likewise, the development of survivorship care programmes and attention to quality of life have increased in recent years, which may have influenced our findings. However, for participants who reported that adequate information was not provided, we verified in which year they were diagnosed to ensure that this was a relatively recent experience. Considering recent developments in care programs, the time since diagnosis is a relevant factor, as treatment and social policy change over time. Therefore, assessment on a large scale, including respondents who have been recently diagnosed, is recommended in future research.

A major strength of our study is the active involvement of AYAs with lived experiences as co-researchers. Throughout all phases of this study, we collaborated with AYAs who were diagnosed with cancer during their higher education. Involving AYAs in research concerning their own experiences is invaluable for gaining in-depth knowledge and for understanding their needs in the context of our research findings. Their involvement enhances the credibility and confirmability and improves the translation to clinical practice, which is well-documented [36,53,54]. Furthermore, involvement as co-researchers empowers AYAs by placing them in a position of relative control, adding their knowledge to the project [55]. Discussions with AYA co-researchers throughout the study improved our understanding and increased the quality of the research, such as by discussing the names and content of the themes, and adapting the language used in the interviews.

### 4.2. Implications for Practice, Policy and Research

Our research contributes to the Dutch AYA Care Network’s goal of improving educational and work-related support [38] and offers guidance on important considerations for the development of these future support programs. In the development of future support programs, it is important to acknowledge both the personal experiences of AYA students—such as the impact of cancer, meaning-making, personalized contextual factors, and adolescent development—and critically examine the country-specific systemic structures influencing their participation in higher education.

The findings of our research have interrelated implications for clinical practice, policy, and future research, and, in order to dedicate attention to all factors, we recommend involving a range of stakeholders in the upcoming steps of research. Ideally, this would include healthcare professionals, education professionals, and policymakers as representatives of the systems involved—healthcare, education, and social structures—alongside AYA patient experts. This multidisciplinary approach increases the likelihood that the support program will be well-aligned with the context [55,56,57]. To enhance the feasibility and reach of future programs, we recommend that the AYA care network take a leading role in their development and implementation. The AYA care network is well positioned for this role, given its specialized expertise, central position in the coordination of care, and commitment to ensuring that every AYA receives specialized, person-centered care. Furthermore, policymakers are the key actors to implement policy change and remove systemic barriers. They should be aware of students’ access to resources and of existing barriers, such as referrals to a psychologist in the university city when a student’s GP is still located near their parents’ home, as well as barriers to accessing financial support, and should actively advocate for reducing these barriers and facilitating access to appropriate support.

At the level of practice, our findings emphasize that healthcare and educational professionals should be aware of specific factors that might put AYAs in vulnerable positions, and how and by whom these should be addressed in a supportive manner. Future research should address their perspectives to gain an in-depth understanding of their professional competence and, in relation to systemic structures, their perceived ownership and responsibility in supporting AYA students. Examining whether barriers arise from knowledge gaps, service gaps, communication issues, or interactions between these factors may help reveal how institutional support pathways and structures could be improved to meet the needs of AYAs.

Another line of future research concerns the use of quantitative approaches to assess the prevalence and impact of a cancer diagnosis on education in larger AYA student populations. This quantitative research could identify factors associated with an increased risk of adverse outcomes for AYA students and examine patterns in effective strategies that AYAs use to self-manage their reintegration into studies or transition to work.

## 5. Conclusions

Dynamic interactions between life stage and health status—and their impact on educational attainment, transition to work, and financial independence—underscores the need to better support younger AYAs and enhance their participation in higher education. This is important both from the individual student’s perspective and from a societal perspective, given the broader socioeconomic consequences. This study aimed to improve the understanding of both the impact on and challenges encountered by AYAs who are diagnosed with cancer during higher education to inform the design of a support program. Although the literature shows that support for AYAs in resuming education and transition to work is essential, our study shows that a clear support pathway is lacking. Evidently, support is not accessible for all AYA students. While every AYA experiences cancer and the challenges that come with it differently, the results highlight commonalities. AYAs face physical, cognitive and mental limitations due to the cancer diagnosis or treatment. Being an AYA student implies age-specific challenges. Their altered abilities, discrepancies between their expectations and reality, and altered identity as a student who experienced cancer may impede the continuation of education or a transition to working life. Additionally, contextual factors such as changing classes, financial impacts, changes in place of residence, and the complexity of navigating healthcare and educational systems play a role in their ability to participate in education and enter the labor market. These personal and contextual factors are important to consider in tailored interventions. However, our results also indicate that systemic structures impede AYAs’ participation and should be addressed. Taken together, our findings underscore the need for coordinated efforts among healthcare providers, educational institutions, and policymakers to support AYAs in their strong drive to re-enter education and to address their unique needs so they can reach their full potential.

## Figures and Tables

**Table 1:** Characteristics of the study population.

Respondents	n = 13
Sex	Female (n = 10; 77%) Male (n = 3; 23%)
Age at time of diagnosis (years)	Range: 19–25 M (SD): 21.3 (2.3)
Age at time of interview (years)	Range: 22–30 M (SD): 26.4 (3.0)
Time since diagnosis (years)	Range: 0–9 M (SD): 4.7 (3.1)
Enrolled in educational level at time of diagnosis	University of applied science (HBO)—(n = 4; 31%) Research university (WO)—bachelor’s program (n = 5; 38%) Research university (WO)—master’s program (n = 4; 31%)
Diagnosis in study year	1 (n = 2, 15%) 2 (n = 4, 30%) 3 (n = 2, 15%) 4 (n = 3, 23%) 5 (n = 2, 15%)
Work status at time of diagnosis	Student and no part-time job (n = 8; 62%) Student and part time job without contract (n = 4; 31%) Student and part time job with contract (n = 1; 8%)
Working hours if working part time	Without contract: Range: 4–19 h/week, M (SD): 10.8 (6.4) With contract: Range: 14 h/week, M (SD): 14 (0)
Living situation *	Student house at time of diagnosis, but returned to parent(s) after diagnosis (n = 5; 38%) Lives with parent(s) at time of diagnosis (n = 3; 23%) Unknown (n = 5; 38%)
Treatment status	In treatment (n = 8; 62%) Finished treatment (n = 5; 38%)
Type of cancer	Brain cancer (n = 1; 8%) Breast cancer (n = 2; 15%) Colorectal cancer (n = 1; 8%) Salivary gland carcinoma (n = 1; 8%) Lymphoid hematological malignancies (Hodgkin) (n = 5; 38%) Male genitalia tumor (n = 1; 8%) Mediastinal germ cell tumor (n = 1; 8%) Thyroid cancer (n = 1; 8%)

* Based on information provided in the interview.

## Data Availability

To protect the confidentiality and privacy of the participants and the conditions of the informed consent, the data underlying this study are not publicly available.

## Abbreviations

The following abbreviations are used in this manuscript:

AYA	Adolescent and Young Adult
CRF	Case Report Form
MBO	Middelbaar Beroepsonderwijs (secondary Vocational Education and Training (VET)
EP	Educational Professional
GP	General Practitioner
HBO	Hoger Beroepsonderwijs (universities of applied sciences)
HCP	HealthCare Professional
WMO	Wet Medisch-wetenschappelijk Onderzoek met mensen (Medical Research Involving Human Subjects Act)
QoL	Quality of Life
SJK	Stichting Jongeren en Kanker (Dutch AYA patient advocacy group)
SRQR	Standards for Reporting Qualitative Research
VET	secondary Vocational Education and Training
WO	Wetenschappelijk Onderwijs (research universities)
